# From Convenience to Clinical Efficacy: Selective TYK2 Inhibition in Psoriasis and the Evolving Role of Next-Generation Oral Targeted Therapies

**DOI:** 10.3390/pharmaceutics18030347

**Published:** 2026-03-11

**Authors:** Klara Andrzejczak, Agata Sternak, Wiktor Witkowski, Bożena Karolewicz, Małgorzata Ponikowska

**Affiliations:** 1 Faculty of Medicine, Wroclaw Medical University, Wybrzeze L. Pasteura 1, 50-367 Wroclaw, Poland; 2 University Centre of General Dermatology and Oncodermatology, Wroclaw Medical University, 50-556 Wroclaw, Poland; 3 Department of Drug Form Technology, Wroclaw Medical University, Borowska 211 A, 50-556 Wroclaw, Poland

**Keywords:** psoriasis, TYK2 inhibitors, deucravacitinib, envudeucitinib, zasocitinib, allosteric inhibition, small-molecule inhibitors, JAK–STAT signaling, IL-23/Th17 axis

## Abstract

Psoriasis is a chronic, immune-mediated inflammatory skin disease requiring effective long-term systemic treatment. Current options, including using conventional small molecules and biological therapies, are limited by adverse events, suboptimal efficacy, or poor adherence due to inconvenient administration. This highlights an unmet need for safe, convenient, and effective oral self-administered dosage form therapies aligned with patient preferences. This review evaluates the mechanism, safety, and efficacy of next-generation tyrosine kinase 2 (TYK2) inhibitors and compares them to currently available therapeutic options. The pathogenesis of psoriasis is driven by chronic systemic inflammation mediated by the interleukin-23 (IL-23)/Th17/interleukin-17 (IL-17) axis. Selective TYK2 inhibitors, such as deucravacitinib, envudeucitinib, and zasocitinib, act through a unique allosteric mechanism by binding to the regulatory pseudokinase domain (JH2) rather than the enzyme’s catalytic domain. This enables highly selective suppression of IL-23-mediated inflammation while mitigating systemic toxicity seen with nonselective Janus kinase (JAK) inhibitors. Clinical trials (POETYK PSO-1 and PSO-2) and long-term extension studies demonstrate that deucravacitinib provides superior efficacy compared to the first-generation oral small molecule apremilast, with high and sustained response rates. It maintains durable efficacy for up to four years in patients with moderate to severe plaque psoriasis and shows a stable long-term safety profile, with low incidence of major adverse cardiovascular events (MACEs), venous thromboembolism (VTE), serious infections, and malignancies. Selective TYK2 inhibition bridges the therapeutic gap, providing an optimal balance of efficacy and oral convenience. With the potential to improve patient adherence and quality of life, these agents represent a promising option to become a first-line oral systemic therapy for psoriasis.

## 1. Introduction

### 1.1. Systemic Nature of Psoriasis and Comorbidities

Psoriasis is a chronic, immune-mediated inflammatory skin disease characterized by well-demarcated, erythematous plaques with silvery-white scales, affecting approximately 2–3% of the global population, which corresponds to nearly 125 million individuals worldwide. The most common clinical form is plaque psoriasis, which accounts for over 80% of all cases and is characterized by a relapsing–remitting course [1,2,3].

Until recently, chronic inflammatory skin diseases such as psoriasis were considered conditions confined to the skin. Current evidence confirms that psoriasis has a complex, systemic nature that extends beyond dermatology. The interleukin-23 (IL-23)/Th17/interleukin-17 (IL-17) axis plays a central role in its pathogenesis, with its sustained activation and elevated levels of pro-inflammatory cytokines forming the basis of generalized inflammation [4,5,6].

This means that psoriasis is recognized as an independent risk factor for comorbidities. Approximately 75% of patients present with at least one comorbidity, which has a significant impact on their overall health [6].

The most commonly observed comorbidities include psoriatic arthritis, metabolic syndrome, and obesity. Regardless of whether the disease is mild or severe, psoriasis is associated with an increased risk of cardiovascular disease, including atherosclerosis and major adverse cardiovascular events such as myocardial infarction and stroke. Greater disease severity further increases the risk of mortality from these causes [7,8,9,10,11,12].

### 1.2. Impact of Psoriasis on Patient Quality of Life and Therapeutic Implications

The chronic and multifaceted nature of psoriasis profoundly affects patients’ daily functioning and health-related quality of life (HRQoL). Burdensome symptoms, comorbidities, and the need for long-term therapy contribute to reduced life satisfaction and a high prevalence of mental health disorders, such as anxiety and depression [13,14,15,16].

In the context of chronic disease, adherence rates often remain suboptimal, and unmet patient needs lead to dissatisfaction with available treatment options. Research indicates that the route of administration is as important to patients as treatment effectiveness. Taking patient preferences into account, in line with the personalized medicine approach, enhances comfort and engagement in the treatment process, which contributes to improved clinical outcomes [15,17,18,19,20].

In patients with moderate to severe disease (Body Surface Area [BSA] ≥ 3%) who require systemic therapy, achieving long-term control of inflammation while maintaining an acceptable safety profile remains a key challenge [21].

### 1.3. Bridging the Gap: The Persisting Unmet Need for Safe and Effective Oral Therapies

Treatment for moderate to severe psoriasis has evolved significantly, from conventional systemic therapies to biologics, which offer high efficacy, although their use is limited by factors such as the need for invasive drug administration in injections and the risk of immunogenicity. Targeted small molecules provide an alternative option, but their initial generations did not always offer sufficient disease control and carried a risk of adverse events.

Despite progress, patient needs remain unmet. Available treatment options still have some limitations in terms of long-term efficacy, tolerability, safety, and mode of administration. In clinical practice, treatment adherence is often insufficient, partly because individual patient preferences, determined by age, comorbidities, duration of psoriasis, and prior treatments, are not always taken into account.

Patients expect a high probability of improvement, optimal skin clearance, a convenient mode of administration, and a favorable safety profile. Research indicates that many patients prefer oral therapy because it is less psychologically challenging. A strong aversion to injections may lead them to accept slightly lower efficacy in exchange for greater treatment comfort. Effective communication with patients and consideration of their preferences can significantly improve treatment adherence and long-term persistence.

In this context, selective tyrosine kinase 2 (TYK2) inhibitors represent a significant step forward. This new generation of oral agents targeting key intracellular signaling pathways offers a potential compromise between the high efficacy of biologic therapies and the convenience of a noninvasive, painless oral administration route, which is often preferred by patients [17,18,22,23,24,25,26,27,28].

### 1.4. Aim of the Review

In light of the unmet need for novel, safe, and highly effective targeted oral therapies, this review aims to examine the unique allosteric mechanism of action, safety profile and clinical efficacy of next-generation TYK2 inhibitors. It also assesses their therapeutic potential relative to current systemic and biologic options, highlighting how highly selective TYK2 targeting may optimize safety while preserving high efficacy and the convenience of oral administration.

## 2. From Conventional Systemics to Precision Therapies: The Evolution of Psoriasis Management

Systemic treatment of moderate to severe psoriasis has changed a lot over the past decades: starting with nonspecific immunosuppressive therapy, moving through the era of biologics to modern targeted oral small molecules [21,29,30].

### 2.1. Conventional Systemic Therapies: Established Oral Options

According to the EuroGuiDerm guidelines, conventional systemic therapies (such as methotrexate, cyclosporine, acitretin, or dimethyl fumarate) are the first-line systemic treatment for patients with moderate to severe psoriasis. The choice of specific therapy depends on the patient’s individual characteristics, including comorbidities and the safety profile, with cyclosporine primarily recommended for short-term induction therapy.

Although these drugs are effective and offer a significant advantage in the form of convenient oral administration, their potential to achieve high remission rates (Psoriasis Area and Severity Index [PASI] 75 or PASI 90) is lower than that of biologic therapies. This limitation is due to their nonselective mechanism of action, which leads to a broader adverse event profile and the need for routine laboratory monitoring.

Biological therapies are recommended in cases of lack of efficacy, intolerance, or contraindications to conventional therapy. In severe cases where conventional therapy is expected to be ineffective, biological therapies may also be considered as first-line therapy [16,21,29,31,32].

### 2.2. Targeted Biologic Therapies: Modulating Key Cytokine Pathways

A better understanding of the pathogenesis of chronic inflammatory skin diseases has allowed for the development of new biological therapies directed against specific cytokines and immune pathways underlying disease development. Targeted monoclonal antibodies modulating cytokines of the Th17-dependent inflammatory axis have revolutionized psoriasis treatment.

The most important biological therapies currently used include drugs targeting tumor necrosis factor (TNF) (adalimumab, etanercept, certolizumab pegol, infliximab), IL-12/23p40 inhibitors (ustekinumab), IL-17A inhibitors (ixekizumab and secukinumab), an IL-17RA antagonist (brodalumab), and IL-23p19 inhibitors (guselkumab, risankizumab, and tildrakizumab) [16,32,33,34,35,36,37]. The group of drugs targeting the IL-17 pathway also includes bimekizumab, a monoclonal antibody simultaneously neutralizing IL-17A and IL-17F, distinguishing it from classical IL-17A inhibitors [38].

Biological therapies have demonstrated high clinical efficacy in patients with moderate to severe psoriasis [39]. This is confirmed by a large Cochrane network meta-analysis by Sbidian et al., which showed that infliximab, bimekizumab, ixekizumab, and risankizumab were most effective in achieving near-complete clearance of skin lesions (PASI 90) compared to placebo [32].

Biologics differ from traditional systemic medications in their route of administration. For some patients, having subcutaneous injections or intravenous infusions as drug dosage forms can be a limitation. Additionally, some biologics may lose efficacy over time or carry a risk of immunogenicity [16,18,39].

### 2.3. The Era of Targeted Small Molecules: The Role of Apremilast (PDE4 Inhibition)

The next step in therapeutic evolution was the introduction of targeted small molecules acting intracellularly. The first agent in this class for the treatment of psoriasis was apremilast, an oral phosphodiesterase 4 (PDE4) inhibitor.

Inhibiting this enzyme increases adenosine 3′,5′-cyclic monophosphate (cAMP) levels, leading to reduced expression of proinflammatory mediators and increased expression of anti-inflammatory cytokines. This mechanism exerts anti-inflammatory effects without the immunosuppressive properties typical of other systemic therapies [23,40,41].

Apremilast has become an alternative treatment, offering convenient oral dosing and a good safety profile, as it does not lead to organ toxicity. Its effectiveness is modest and it can cause gastrointestinal side effects such as diarrhea and nausea.

The efficacy of apremilast remains suboptimal compared with biologics, leaving a therapeutic gap for patients looking for higher efficacy while maintaining oral administration [23,30,40,41,42,43,44,45,46,47].

The limitations of currently available therapies have prompted the search for new approaches, targeting key signaling pathways such as the Janus kinase (JAK)–signal transducer and activator of transcription (STAT) pathway, whose inhibition underlies the action of a new generation of selective TYK2 inhibitors, potentially providing significant therapeutic benefits [48].

## 3. Mechanism of Action and the Role of TYK2 in the Pathogenesis of Psoriasis

### 3.1. Key Cytokine Pathways in Psoriasis Pathogenesis and Their Involvement in JAK/STAT Signaling

#### 3.1.1. The IL-23/Th17/IL-17 Axis in Keratinocyte Hyperproliferation and Inflammation

The pathogenesis of psoriasis, an immune-mediated inflammatory disease (IMID), is driven by chronic inflammation, maintained by components of both the innate and adaptive immune systems. Disease progression is influenced by genetic factors, including the HLA-C*06:02 risk allele, which can interact with a variant in the *ERAP1* gene. Disease development is also affected by environmental factors. Epidermal antigens activate dendritic cells in the dermis, which in turn secrete proinflammatory cytokines, including IL-23.

The differentiation of naive T cells into Th17 cells requires interleukin-6 (IL-6), interleukin-1β (IL-1β), and transforming growth factor-β (TGF-β), whereas IL-23 plays a crucial role in maintaining and stabilizing the Th17 phenotype and their pathogenic activity. Activated Th17 cells produce IL-17A, IL-17F, IL-22, TNF-α, and other inflammatory mediators, driving keratinocytes toward uncontrolled proliferation and impaired differentiation, resulting in the characteristic psoriatic lesions. These mediators also stimulate keratinocytes to produce cytokines and chemokines, which recruit and activate cells of the innate immune system, thereby amplifying the inflammatory response.

The IL-23/IL-17 pathway represents a central mechanism in the pathogenesis of psoriasis, forming a self-perpetuating cycle of chronic inflammation. Signaling of numerous key cytokines within this inflammatory axis is dependent on the JAK–STAT pathway [5,49,50,51].

#### 3.1.2. Structure and Function of the JAK–STAT Signaling Pathway

JAK and STAT signaling pathways play a crucial role in transmitting cytokine signals within cells. They regulate immune function, as well as cell proliferation, differentiation, and apoptosis, and are also involved in hematopoiesis and lipid metabolism [50,52].

The JAK family comprises four tyrosine kinases: JAK1, JAK2, JAK3, and TYK2. The STAT factor family consists of seven proteins: STAT1, STAT2, STAT3, STAT4, STAT5a, STAT5b, and STAT6 [39,49,53].

**Figure 1:**
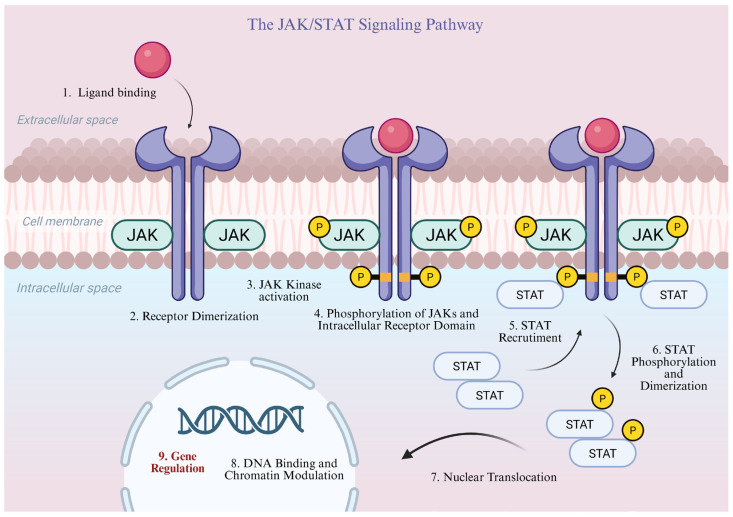
The Janus kinase (JAK)–signal transducer and activator of transcription (STAT) signaling pathway: structure and key steps. The JAK–STAT pathway mediates signal transduction from the cell membrane to the nucleus. Cytokine binding to type I or type II receptors induces receptor dimerization and activation of receptor-associated JAKs (JAK1, JAK2, JAK3, and tyrosine kinase 2 [TYK2]). Activated JAKs phosphorylate each other and the intracellular receptor domains, enabling recruitment and phosphorylation of cytoplasmic STAT proteins. Phosphorylated STATs dimerize and translocate to the nucleus to regulate gene transcription. Different combinations of JAKs mediate signaling for distinct cytokines. Created in BioRender. Andrzejczak, K. (2026) https://BioRender.com/ednmo1s. (accessed on 5 March 2026).

The JAK–STAT signaling pathway mediates signal transduction from the cell membrane to the nucleus and is utilized by numerous cytokines and growth factors. A central component of this system is the non-receptor JAK tyrosine kinases, which are associated with the cytoplasmic domains of type I and II cytokine receptors. Pathway activation occurs upon binding of a specific ligand (cytokine) to the receptor, inducing its dimerization. Receptor-associated JAKs become activated, phosphorylating each other as well as the intracellular domain of their receptor. This allows the recruitment and binding of cytoplasmic STAT proteins. Upon receptor binding, these proteins are phosphorylated, dimerize, and subsequently translocate into the nucleus via Ran-dependent nuclear import. Within the nucleus, activated STAT dimers can directly bind to specific DNA sites and modulate chromatin structure, thereby regulating the transcription of target genes. A schematic representation of these key steps in the JAK–STAT signaling pathway is illustrated in Figure 1. Many cytokines, including IL-2, IL-6, IL-12, IL-15, and IL-23, as well as interferons, signal through the JAK–STAT pathway, whereas others, such as IL-1, IL-17, and tumor necrosis factor TNF, do not [39,52,53,54,55,56,57].

Within the cell, two receptor chains typically associate with distinct JAKs, forming heterodimers such as JAK1/JAK2, JAK1/JAK3, JAK1/TYK2, or JAK2/TYK2. An exception is provided by hematopoietic growth factor receptors, which employ JAK2 homodimers. Each JAK dimer participates in signal transduction for specific cytokines [53,54].

The JAK–STAT pathway is involved in the pathogenesis of numerous chronic immune-mediated inflammatory diseases (IMIDs), including psoriasis, rheumatoid arthritis, and inflammatory bowel disease. In psoriasis, activation of this pathway induces the proliferation of Th17 lymphocytes, keratinocytes, and gamma-delta T cells, underscoring its significance as a key therapeutic target [49,50,52,56].

#### 3.1.3. TYK2 Kinase in IL-23/Th17-Mediated Psoriasis Pathogenesis

TYK2 is an intracellular enzyme in the JAK family that mediates signaling through cytokine receptors involved in immune responses and inflammatory processes. It plays a particularly important role in psoriasis pathogenesis by participating in cytokine signaling that drives excessive Th17 lymphocyte activity, resulting in chronic inflammation and enhanced keratinocyte proliferation, which lie at the core of the disease [50,58,59,60].

**Figure 2:**
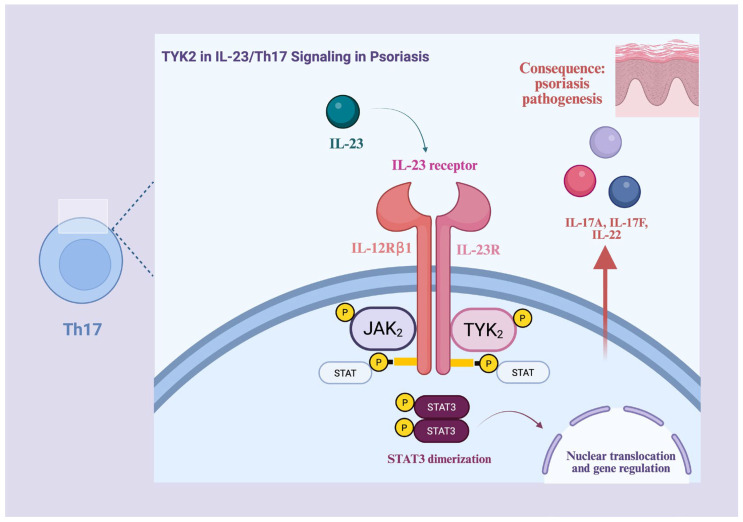
Role of TYK2 signaling in the IL-23/Th17 axis and psoriasis pathogenesis. The IL-23 receptor, composed of IL-12Rβ1 and IL-23R subunits, binds IL-23, leading to activation of the JAK2/TYK2 heterodimer. This triggers phosphorylation and dimerization of STAT3. The resulting STAT3 dimers translocate to the nucleus and regulate target genes, including proinflammatory Th17 cytokines (IL-17A, IL-17F, IL-22). This signaling pathway drives Th17 lymphocyte activity and chronic inflammation, which are key features of psoriasis pathogenesis. Created in BioRender. Andrzejczak, K. (2026) https://BioRender.com/y5ju4cu. (accessed on 5 March 2026).

Functionally, TYK2 forms heterodimers with JAK1 or JAK2: the JAK1/TYK2 heterodimer mediates IL-22, IL-10, and IFN-α/β signaling, whereas JAK2/TYK2 is responsible for IL-12 and IL-23 signaling. The IL-23 receptor consists of the IL-12Rβ1 and IL-23R subunits, with IL-12Rβ1 serving as the shared subunit for both the IL-12 and IL-23 receptors. Upon binding of IL-23 to its receptor, the JAK2/TYK2 heterodimer becomes activated, leading to phosphorylation of STAT3, the principal transducer of IL-23 signaling. STAT dimers then translocate to the nucleus, where they regulate the expression of target genes, including proinflammatory Th17 cytokines such as IL-17A, IL-17F, IL-22, and TNF-α. STAT4 is also activated, although to a much lesser extent. This signaling pathway is illustrated in Figure 2 [48,49,50,53,58,61,62,63,64,65].

TYK2 is crucial for pro-inflammatory IL-23 signal transduction in psoriasis, while it also participates in the IL-10 and IL-13 pathways, which have potential protective effects, highlighting the complexity of this kinase’s function [49].

Its central role in the IL-23/Th17 axis makes it an attractive and biologically justified therapeutic target. Importantly, genome-wide association studies (GWASs) have identified naturally occurring loss-of-function variants in the *TYK2* gene. People with these variants have a much lower risk of developing immune-related diseases, including psoriasis, and do not have a higher risk of viral infections or malignancies. This strongly supports that selective TYK2 inhibition can achieve high therapeutic efficacy while maintaining a favorable safety profile [39,61,66,67].

### 3.2. Molecular Basis of TYK2 Selectivity: Allosteric vs. Orthosteric Mechanisms

Targeting kinases involved in the immune response remains a central research objective due to their crucial role in regulating cellular processes. Given the high sequence and structural similarity among JAK family tyrosine kinases, the innovative and selective mechanism of action of the new generation of oral small-molecule inhibitors is particularly relevant [68,69,70,71].

JAKs consist of four characteristic structural domains: an N-terminal FERM domain and an SH2-type domain, which facilitate the kinase’s binding to the cytokine receptor; a pseudokinase domain (JH2), which plays a key regulatory role; and a C-terminal catalytic domain (JH1), also known as the kinase domain, which is responsible for its enzymatic activity [60,72,73,74,75,76].

JAK inhibitors are divided into two types: allosteric inhibitors, which bind to a site other than the active site and thereby indirectly block the enzyme’s activity by altering its conformation, and orthosteric inhibitors, which are ATP-competitive and directly compete for the binding site within the catalytic domain [39,77].

The active catalytic domain of JH1 exhibits high structural similarity to the adenosine triphosphate (ATP) binding site across the JAK family (JAK1, JAK2, JAK3, TYK2). Classical small-molecule JAK inhibitors act orthosterically, binding to the active site of the JH1 domain and directly competing with ATP for this site. This effectively blocks kinase activity, preventing phosphorylation and subsequent signal transduction. Due to the highly conserved nature of this site, these drugs may also interact with other JAKs, leading to off-target inhibition and associated adverse effects. Classical inhibitors include first-generation drugs, such as tofacitinib (a nonselective pan-JAK inhibitor) and ruxolitinib (selective for JAK1/2), as well as second-generation inhibitors, such as filgotinib, upadacitinib, and abrocitinib, which are selective for JAK1 [39,71,72,73,76].

A new generation of drugs utilizing an allosteric mechanism has emerged to address the limitations of classical inhibitors. Their leading representative is deucravacitinib, a highly selective, oral, small-molecule TYK2 inhibitor that binds to a unique regulatory domain (the JH2 pseudokinase) rather than the enzyme’s active catalytic domain. This drug exhibits over 100-fold greater selectivity for TYK2 compared to JAK1/JAK3, and over 2000-fold greater selectivity compared to JAK2 [50,71,78].

Structural studies have revealed the unique ability of deucravacitinib to inhibit TYK2 kinase in three distinct functional states: an autoinhibited state and two activated states for autophosphorylation and phosphorylation of effector proteins. This mechanism extends beyond a simple steric blockade. By binding to the pseudokinase domain (JH2), the drug stabilizes it in a conformational state that reinforces the natural autoinhibitory interactions with the catalytic domain, effectively locking the enzyme in an inactive state.

Blocking the kinase prevents its activation by the receptor, thereby inhibiting further signaling of proinflammatory cytokines (IL-12, IL-23, and type I interferons).

**Figure 3:**
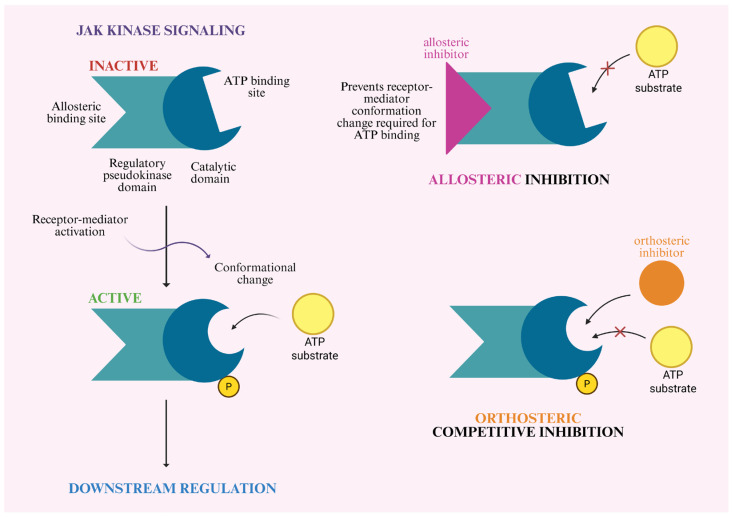
Activation of Janus kinase (JAK) and comparison of non-selective orthosteric JAK inhibition versus selective allosteric tyrosine kinase 2 (TYK2) inhibition. JAKs contain a catalytic domain (JH1) and a regulatory pseudokinase domain (JH2). JAK inhibitors are divided into two types. Classical orthosteric JAK inhibitors bind to the JH1 domain, competing with ATP and blocking kinase activity, which, due to the highly conserved nature of this site, may affect other JAKs. Selective allosteric TYK2 inhibitors bind to the JH2 domain, stabilizing it in a conformational state that effectively locks the enzyme in an inactive state. This prevents kinase activation by the receptor and selectively inhibits signaling of proinflammatory cytokines. Created in BioRender. Andrzejczak, K. (2026) https://BioRender.com/k2tfkmw. (accessed on 5 March 2026).

Figure 3 summarizes the activation of Janus kinases and compares non-selective orthosteric JAK inhibition with the selective allosteric TYK2 mechanism.

Through this selective mechanism, deucravacitinib reduces the expression of genes crucial to the pathogenesis of psoriasis, representing a promising targeted therapy that combines a favorable safety profile with the convenience of oral administration. The therapeutic potential of this mechanism has been confirmed in clinical trials, demonstrating the efficacy of deucravacitinib in the treatment of moderate to severe psoriasis [28,39,50,68,70,78,79,80,81,82,83].

The innovative nature of this new generation of oral targeted therapies is further highlighted by the development of additional molecules, such as envudeucitinib. This is another highly selective oral TYK2 inhibitor, whose efficacy and safety profile are currently being evaluated in clinical trials [84].

## 4. Advances in Safety of Targeted Therapies: Selective TYK2 vs. Nonselective JAK Inhibitors

### 4.1. Safety Profile of Nonselective JAK Inhibitors: Clinical and Mechanistic Insights

With the rapid development of new therapeutic strategies, JAK inhibitors have attracted considerable interest as immunomodulatory agents for a variety of chronic inflammatory diseases, including psoriasis. However, initial optimism regarding their efficacy has been tempered by safety concerns arising from the nonselective mechanism of action of these small molecules [85,86,87,88].

#### 4.1.1. Mechanistic Basis of Toxicity in Nonselective JAK Inhibition

Individual kinases of the JAK family perform key physiological functions, and their nonselective blockade can lead to off-target toxicity, significantly limiting the safety profile of this class of drugs.

For example, JAK2 kinase is essential for normal hematopoiesis (erythropoiesis, myelopoiesis, and thrombopoiesis). Because hematopoietic cytokines, including erythropoietin, signal through JAK2, its inhibition can directly increase the risk of anemia and other cytopenias. JAK2 is also involved in leptin signaling, which may contribute to disturbances in lipid and glucose metabolism.

JAK1- and JAK3-dependent pathways are responsible for lymphocyte homeostasis and for the development and function of natural killer (NK) cells, which are crucial for antiviral and antitumor defense. Importantly, interferon-γ (IFN-γ) and IL-15 signaling, key mediators of antiviral immunity, are mediated by JAK1, JAK2, and JAK3. Blocking these pathways explains the increased risk of viral infections, including Herpes Zoster, and is likely a common effect of all nonselective JAK inhibitors [89,90,91,92,93,94].

#### 4.1.2. ORAL Surveillance Trial: Clinical Evidence of Safety Risks

The Oral Rheumatoid Arthritis Trial (ORAL) Surveillance study was pivotal in evaluating the safety of nonselective JAK inhibitors. It was a randomized, open-label, non-inferiority study conducted as a Phase 3b/4 post-marketing trial.

The study included patients with active rheumatoid arthritis despite methotrexate therapy, aged ≥50 years, and with at least one additional cardiovascular risk factor. The objective was to evaluate the noninferiority of tofacitinib compared with TNF inhibitors for major adverse cardiovascular events (MACEs) and malignancies (excluding nonmelanoma skin cancer, NMSC). Failure to meet the noninferiority criteria highlighted the limitations of tofacitinib’s safety profile.

Further post hoc analyses of the ORAL Surveillance study allowed for a more detailed examination of the relationship between the use of nonselective JAK inhibitors and the occurrence of serious adverse events, and enabled the identification of a population at high risk for these complications [95,96,97,98].

#### 4.1.3. Regulatory Warnings and Guideline Recommendations

As a result, the U.S. Food and Drug Administration (FDA) issued a Boxed Warning, the highest level of warning, indicating an increased risk of MACEs, malignancies (particularly lymphomas and lung cancer), thrombosis, serious infections, and death associated with tofacitinib and other nonselective JAK inhibitors [99].

The European Medicines Agency (EMA) also recommended avoiding the use of these drugs as first-line therapy for inflammatory and dermatological diseases in patients with risk factors. This group included individuals aged 65 years and older, smokers, and patients at increased risk of serious cardiovascular events or malignancies. The observed risks were considered to result from nonselective inhibition of JAKs [100].

These warnings are based primarily on the results of the ORAL Surveillance study in a population of patients with rheumatoid arthritis. Extrapolating the findings of this study to patients with dermatological conditions requires further research and long-term safety evaluation, which nevertheless fully warrants heightened caution [95,98,101,102].

Other identified adverse events potentially related to the nonselective mechanism of action of classical JAK inhibitors include hematological changes, hyperlipidemia, gastrointestinal perforation, and hepatic and renal dysfunction, which further complicate the safety profile of this class of drugs [50].

#### 4.1.4. Safety Limitations of Nonselective JAK Inhibitors: Deucravacitinib Fills the Therapeutic Gap

Although tofacitinib demonstrated significant efficacy in treating moderate to severe plaque psoriasis in Phase III clinical trials and offered a convenient oral formulation, its serious safety concerns proved unacceptable. As a result, the FDA rejected the approval application, concluding that the benefit–risk ratio was unacceptable [85,86,103].

Safety concerns with nonselective JAK inhibitors had prevented their approval for treating plaque psoriasis, creating a significant gap in access to safe oral therapies, which was addressed by deucravacitinib. This drug now provides a safe and effective alternative to previously available psoriasis treatments [104,105].

### 4.2. Safety and Tolerability of Selective TYK2 Inhibitors

#### 4.2.1. The Impact of Selectivity on the Safety Profile

Deucravacitinib’s high selectivity for TYK2 kinase directly enables precise modulation of inflammatory pathways. By allosterically stabilizing the enzyme in its inactive state, the drug selectively suppresses cytokine signaling pathways critical to psoriasis pathogenesis, including IL-23 and type I interferons. Consequently, the drug effectively controls inflammatory processes and keratinocyte hyperproliferation [78,106].

The selectivity of deucravacitinib for TYK2 kinase was confirmed in a randomized controlled trial (RCT) conducted by Catlett et al. In a pharmacodynamic analysis involving patients with moderate to severe psoriasis, the drug’s effects on biomarkers of the IL-23/TH17 pathway and type I interferons were evaluated, while laboratory parameters indicative of JAK1–3 kinase activity were monitored to verify deucravacitinib’s selectivity for TYK2.

The analysis included hemoglobin concentration and platelet count (JAK2-dependent biomarkers), total cholesterol, triglycerides, and creatine kinase (JAK1–3-dependent), as well as neutrophil levels. Additionally, T cell, B cell, and NK cell counts (JAK1/JAK3-dependent) were evaluated using flow cytometry. The results demonstrated that JAK1–3-dependent biomarkers remained unaffected by deucravacitinib. The fact that these specific markers were unaffected, while disease-related pathways were effectively suppressed, provides strong evidence that deucravacitinib selectively targets TYK2.

Despite a duration of only 12 weeks, the study provided key molecular evidence that is supported by long-term study results [80].

The unique safety profile resulting from this selectivity makes deucravacitinib a competitive alternative to existing systemic therapies, effectively filling a therapeutic gap.

#### 4.2.2. Comprehensive Evaluation of the Long-Term Safety of Deucravacitinib

The results published by Armstrong et al. provided key evidence for the long-term and favorable safety profile of deucravacitinib, the first-in-class selective TYK2 inhibitor. This evidence was based on an integrated analysis of data from two pivotal randomized phase III trials (POETYK PSO-1 and POETYK PSO-2) and a non-randomized, open-label Long-Term Extension (LTE), which enabled a comprehensive assessment of long-term safety in 1519 patients with moderate to severe plaque psoriasis who received at least one dose of the drug over 3 years.

Safety assessment was conducted by monitoring adverse events (AEs), serious AEs (SAEs), deaths, AEs of interest (AEIs), and AEs leading to discontinuation.

A central focus was placed on adverse events of interest (AEIs), which were defined based on comorbidities (e.g., cardiovascular), the drug’s tolerability profile in phase 1–2 studies, and adverse events associated with other approved psoriasis therapies.

Adverse events of interest (AEIs) included serious infections and selected viral infections (including herpes zoster and COVID-19), MACEs, venous thromboembolism (VTE), malignancies, and selected dermatological events such as acne and folliculitis. Detailed laboratory parameters were also assessed, including hematological, biochemical, and lipid parameters.

The key safety indicator was the exposure-adjusted incidence rate (EAIR), expressed per 100 person-years. The analysis showed that EAIRs for overall AEs, SAEs, and treatment discontinuations were lower or comparable to those observed in the first year of treatment over the 3-year cumulative follow-up, indicating that the risk did not increase with longer-term treatment.

EAIRs for events of special interest, including MACE, VTE, and malignancies, remained low and stable over time, with no upward trend.

The comprehensive assessment of the long-term safety of selective TYK2 inhibitors is complemented by an analysis of the overall tolerability profile, focusing on the most frequently observed adverse events (EAIR ≥ 5 per 100 person-years). The most commonly reported events were nasopharyngitis, upper respiratory tract infections, and COVID-19.

Notably, COVID-19 cases increased over the three years, peaking during the global pandemic, whereas the incidence of other infections remained comparable to or lower than in the incidence the first year of treatment. General and gastrointestinal symptoms, including headache, joint pain (arthralgia), and diarrhea, were also characteristic of TYK2 inhibitor therapy, but they rarely led to treatment discontinuation. Importantly, the overall rate of treatment discontinuation due to adverse events decreased compared with the first year of treatment (from 4.4 to 2.4 per 100 person-years), confirming the sustained good tolerability of the drug. A detailed summary of EAIR values for adverse events (AEs) over the three-year follow-up period is presented in Table 1.

During follow-up, no clinically significant changes from baseline or abnormal trends were observed in hematologic (blood counts), biochemical (ALT, AST, creatinine, creatine kinase), or lipid (total cholesterol) parameters. In most patients, these parameters remained within normal limits throughout the study period, confirming the high selectivity of deucravacitinib. A small, clinically insignificant increase in triglycerides was observed, without a corresponding rise in the atherogenic LDL fraction. This effect is not specific to TYK2 inhibitors and may also occur with other treatments, such as TNF inhibitors or cyclosporine, or as a consequence of metabolic complications associated with psoriasis itself [106].

No differences in the safety profile of deucravacitinib were noted with respect to age, sex, race, body weight, geographic region, or prior psoriasis therapy [107].

The results of this integrated analysis demonstrate that deucravacitinib has a consistent safety and tolerability profile. The three-year cumulative follow-up, consistent with previous studies in patients with moderate-to-severe plaque psoriasis, suggests that deucravacitinib represents a competitive option among currently available systemic therapies [106].

Recent reports covering up to four years of follow-up confirm the long-term stability and favorable safety profile of deucravacitinib [59].

## 5. Clinical Efficacy and Therapeutic Value of Next-Generation Oral Targeted Therapies

### 5.1. Deucravacitinib: The First-in-Class Selective TYK2 Inhibitor

#### 5.1.1. Regulatory Status, Administration, and Dosing of Deucravacitinib

Deucravacitinib is an orally administered, selective TYK2 inhibitor and the first allosteric inhibitor in its class approved for clinical use. Developed by Bristol Myers Squibb, it was first authorized by the FDA in the United States on 9 September 2022, for adults with moderate to severe plaque psoriasis who are candidates for systemic therapy or phototherapy [32,104,108,109]. Shortly thereafter, on 26 September 2022, it was approved in Japan for plaque psoriasis, generalized pustular psoriasis, and erythrodermic psoriasis [104,107,108], and in March 2023, the European Medicines Agency (EMA) approved it in the EU for moderate to severe psoriasis [108].

Deucravacitinib is also under investigation for other immune-mediated diseases, including lupus and inflammatory bowel diseases, with ongoing clinical studies evaluating its efficacy and safety worldwide [104].

The recommended dosage for adults with moderate to severe plaque psoriasis is 6 mg once daily, taken with or without food. Concomitant use with other potent immunosuppressants is not recommended. Prior to therapy, patients should be screened for active or latent tuberculosis, and appropriate anti-tuberculosis treatment initiated if needed. No dose adjustment is required for mild or moderate hepatic impairment; however, the drug is not recommended in severe hepatic impairment (Child–Pugh class C) and is contraindicated in patients with known hypersensitivity [28,110,111].

#### 5.1.2. Clinical Efficacy of Deucravacitinib: From Phase 1 to Phase 3

Clinical research on deucravacitinib began with a Phase 1 trial involving 108 healthy participants, who were randomized to receive either deucravacitinib or a placebo. The study demonstrated that the drug was safe and generally well-tolerated. The incidence of adverse events was similar in both groups, with the most frequently reported events including headache, nausea, rash, and upper respiratory tract infections [66,112].

In the Phase 2 clinical trial, 267 patients with moderate to severe psoriasis were enrolled and assigned to six treatment groups receiving one of the following regimens: 3 mg of deucravacitinib every other day, 3 mg once daily, 3 mg twice daily, 6 mg twice daily, 12 mg once daily, or placebo. At week 12, the proportion of patients achieving PASI 75 (a 75% reduction in disease severity) was higher in all deucravacitinib groups compared with placebo. For the highest dose (12 mg once daily), 75% of patients achieved PASI 75. Detailed results for all dose groups are presented in Table 2. These findings demonstrate a clear dose-dependent increase in treatment efficacy [66,113].

Improvement in patients’ quality of life was evaluated using the Dermatology Life Quality Index (DLQI). Patients who achieved greater clinical improvement in psoriasis symptoms reported corresponding improvements in quality of life; however, complete skin clearance was not required to reach a DLQI score of 0/1, indicating no impact on daily functioning [66,114].

Adverse events occurred in 51% of patients receiving placebo and in 55–80% of those treated with deucravacitinib, with the highest incidence observed in the 6 mg twice-daily group. The most common adverse events were nasopharyngitis, headache, diarrhea, nausea, and upper respiratory tract infections [66,113].

Subsequently, two large Phase 3 clinical trials were conducted: POETYK PSO-1 and POETYK PSO-2 [66,107,111,115]. These global studies were randomized, double-blind, double-dummy, and placebo-controlled, including an active comparator [28,107,116,117]. POETYK PSO-1 (NCT03624127) was conducted across 165 sites, while POETYK PSO-2 (NCT03611751) included 205 sites worldwide.

The trials enrolled adults aged 18 years or older with moderate to severe psoriasis. Eligible patients had a Physician’s Global Assessment (sPGA) score of ≥3, a PASI score of ≥12, involvement of at least 10% of the body surface area, and a disease duration of at least 6 months prior to screening.

In total, 1683 patients participated in the POETYK PSO-1 and POETYK PSO-2 studies. In both trials, participants were randomized in a 2:1:1 ratio to receive deucravacitinib 6 mg once daily, apremilast 30 mg twice daily, or placebo [28,107,116,117].

The study lasted 52 weeks and included a 4-week screening period, followed by a 16-week placebo-controlled treatment phase with deucravacitinib and apremilast (weeks 0–16). After this phase, treatment with apremilast and deucravacitinib was continued for an additional 8 weeks (weeks 16–24). Patients in the placebo group crossed over to deucravacitinib at week 16. Participants receiving deucravacitinib who achieved a PASI 75 response at week 24 were re-randomized 1:1 to either continue deucravacitinib or switch to placebo for the remainder of the study (weeks 24–52). Efficacy assessments were conducted at weeks 1, 2, 4, 8, 12, and 16, and subsequently every 4 weeks from week 16 through week 52 [28,107,111,117]

After completing the study, patients were eligible to enter the long-term extension (LTE) study POETYK, where they could continue treatment for up to an additional 3 years to evaluate the long-term effects of deucravacitinib [83,111,116].

In both the POETYK PSO-1 and PSO-2 studies, deucravacitinib demonstrated superior efficacy compared with apremilast and placebo [28,117,118]. At week 16, a higher proportion of patients treated with deucravacitinib achieved a PASI 75 response compared with those receiving apremilast or placebo. Similarly, more patients in the deucravacitinib group reached an sPGA score of 0/1 than in the apremilast or placebo groups. Detailed percentage data are provided in Table 3 [28,117,118].

In the POETYK PSO-2 study, 80.4% of patients who responded to deucravacitinib and continued treatment from week 24 to week 52 maintained their PASI 75 response. The median time to loss of PASI 75 response was approximately 85 days (12 weeks) [117,118].

Data from the open-label extension phase indicate that the efficacy of deucravacitinib may be maintained for up to 2 years [118].

#### 5.1.3. Time to Clinical Response and Long-Term Efficacy of Deucravacitinib

In the PSO-1 study, a significant clinical response was observed as early as week 16. PASI 75 was achieved by 58.4% of patients, and sPGA 0/1 by 53.6%. This clinical response was maintained over 52 weeks of treatment with deucravacitinib [28,111].

Patients who completed the PSO-1 and PSO-2 trials and participated in the long-term POETYK LTE study received 6 mg of deucravacitinib daily. The LTE study demonstrated that patients maintained efficacy for up to 3 years (148 weeks), with a safety profile consistent with that observed during the first year [83,111,116]. Moreover, the incidence of adverse events was low and remained stable or even decreased over the 3-year period [116]. Overall, deucravacitinib demonstrates sustained efficacy over a 4-year treatment period, with a consistent safety profile [59]. The duration of response after treatment discontinuation was also assessed: the median time to loss of PASI 75 response was approximately 85 days (12 weeks) [28,118].

### 5.2. Envudeucitinib: A Highly Selective TYK2 Inhibitor

#### 5.2.1. Administration and Dosing of Envudeucitinib

Envudeucitinib is another highly selective TYK2 inhibitor, administered orally at a dose of 40 mg once or twice daily. Currently being investigated for the treatment of moderate-to-severe plaque psoriasis, it shows promising potential for long-term use, including in patients with comorbidities. Phase 3 clinical trials are ongoing to confirm its efficacy and safety profile [84].

#### 5.2.2. Clinical Efficacy of Envudeucitinib: Evidence from Clinical Trials

Envudeucitinib (formerly ESK-001) was evaluated in patients participating in the STRIDE study, a Phase 2, double-blind clinical trial lasting 16 weeks. The study included a 4-week screening period, a 12-week treatment period, and a 4-week follow-up. A total of 288 patients were enrolled across 30 sites in North America and Europe. Participants were randomly assigned to six groups to receive either placebo or envudeucitinib at doses of 10 mg, 20 mg, or 40 mg once daily, or 20 mg and 40 mg twice daily.

Envudeucitinib demonstrated dose-dependent efficacy in improving psoriasis severity and patient-reported quality of life. At all dose levels, a higher proportion of patients achieved PASI 75 compared with placebo, with the greatest efficacy observed in the 40 mg twice-daily group. Detailed data, including percentage values and dosing information, are presented in Table 4. In the highest-dose group, clinical responses were maintained during the 4-week follow-up period, with 83% of patients who achieved PASI 75 at week 12 maintaining this response after treatment discontinuation [119].

Envudeucitinib was generally safe and well-tolerated. Only 2.6% of participants discontinued treatment due to adverse events. The most frequently reported adverse events were headache, upper respiratory tract infections, and nasopharyngitis, while less common events included acne and folliculitis. The highest incidence of adverse events (23%) was observed in the group receiving the highest dose of the drug. Across all treatment groups, adverse events were predominantly mild or moderate in severity. No deaths, cases of tuberculosis, herpes zoster, opportunistic infections, or oral ulcers were reported [119].

Patients who completed the STRIDE study were eligible to participate in the OLE study, an ongoing Phase 2 trial evaluating the long-term safety and efficacy of envudeucitinib. A total of 165 patients from the original study were assigned to receive envudeucitinib at 40 mg once or twice daily, depending on their previous STRIDE dose; patients previously assigned to the placebo group were also enrolled. One patient was excluded after being diagnosed with coronary artery obstruction at week 16 of STRIDE (day 1 of OLE). Between weeks 40 and 64, all patients were switched to the 40 mg twice-daily dose due to its optimal efficacy and favorable benefit–risk profile [84].

Safety and efficacy assessments were performed on day 1 and at weeks 2, 4, 8, 12, and 16, and subsequently every 12 weeks. At week 52, 125 patients were still receiving treatment. Only 3.7% of participants discontinued due to adverse events, with the remainder discontinuing for other reasons. Treatment over the full 52-week period was generally safe. Overall, 65.9% of patients experienced at least one adverse event. The most frequently reported events included nasopharyngitis, upper respiratory tract infections, headache, and COVID-19 infections, while acne and folliculitis occurred less frequently. No dose-dependent effect was observed on the overall incidence of adverse events (AEs) or on the incidence of the most common treatment-emergent adverse events (TEAEs).

Efficacy results confirmed dose-dependent effects similar to those observed in the STRIDE trial. After 52 weeks of treatment, patients receiving 40 mg twice daily demonstrated marked and sustained improvements in both PASI 75 and sPGA 0/1 endpoints, supporting the long-term therapeutic potential of envudeucitinib [84]. Detailed data are presented in Table 5.

#### 5.2.3. Time to Clinical Response and Long-Term Efficacy of Envudeucitinib

In the STRIDE study, envudeucitinib demonstrated dose-dependent efficacy at week 12 [119]. Among patients receiving 40 mg twice daily, 64% achieved PASI 75, and 59% achieved an sPGA score of 0/1. Following discontinuation of the 40 mg twice-daily regimen, 83% of patients who had achieved PASI 75 at week 12 maintained their therapeutic response during the 4-week follow-up period [119]. The long-term OLE study confirmed that envudeucitinib was well-tolerated, with a safety profile consistent with earlier findings. During long-term treatment, the most favorable outcomes were observed in patients receiving 40 mg twice daily, and these effects remained stable over time [84].

### 5.3. Zasocitinib: Another Selective TYK2 Inhibitor

#### 5.3.1. Administration and Dosing

Zasocitinib is an allosteric TYK2 inhibitor with a mechanism of action analogous to that of deucravacitinib. It has been bioengineered to demonstrate enhanced selectivity for the TYK2 JH2 domain. Administered orally, it has shown high efficacy in clinical trials and is associated with a favorable safety profile [67,120].

#### 5.3.2. Clinical Efficacy of Zasocitinib: Evidence from Clinical Trials

Zasocitinib was evaluated in a randomized, double-blind, placebo-controlled Phase 2b trial that included 259 patients with moderate to severe plaque psoriasis. Patients were randomized in a 1:1:1:1:1 ratio to receive 2 mg, 5 mg, 15 mg, or 30 mg of the drug once daily, or placebo. The study lasted 12 weeks, followed by an additional 4-week observation period [67].

At week 12, zasocitinib showed a clear dose-dependent effect, with all active treatment groups achieving better responses than placebo. The highest-dose group showed the strongest clinical improvements, including PASI 90 and PASI 100 responses [67]. Detailed data, including percentage values, are presented in Table 6.

Adverse events were reported in 53–62% of patients treated with zasocitinib, compared with 44% in the placebo group. The most frequently reported adverse events were COVID-19, acne/acne dermatitis, and diarrhea. No dose-dependent relationship was observed for specific adverse events. Two serious adverse events (SAEs) were reported, and only 2–4% of patients discontinued treatment due to adverse events. Currently, two Phase 3 studies are ongoing, involving longer treatment durations and larger patient populations [67].

#### 5.3.3. Time to Clinical Response and Long-Term Efficacy of Zasocitinib

In the study evaluating zasocitinib, the first treatment effects were observed as early as week 4 and were maintained throughout the 12-week treatment period. Ongoing Phase 3 trials will provide further data on the long-term efficacy and safety of zasocitinib [67].

### 5.4. Comparing Efficacy and Safety of TYK2 Inhibitors with Established Therapies

#### 5.4.1. Comparison with First-Generation Targeted Oral Therapies

To establish the clinical positioning of TYK2 inhibitors (e.g., deucravacitinib) in psoriasis management, it is important to compare them with existing oral therapies, such as apremilast. Apremilast inhibits phosphodiesterase-4 (PDE4) and modulates the immune system by increasing intracellular cAMP and reducing the production of IL-2, IL-8, IFN-γ, and TNF, and is approved for the treatment of psoriasis [121,122].

Comparison of these two drugs can be based on the POETYK PSO-1, PSO-2, and long-term extension (LTE) studies, in which some patients received apremilast, others received deucravacitinib, and the remaining patients received placebo [28,107,116,117]. Based on the POETYK trials, a significantly higher treatment response was observed at week 16 in patients treated with deucravacitinib compared with apremilast [28,117].

In the POETYK PSO-1 study, PASI 75 was achieved by 58.4% of patients receiving deucravacitinib, compared with 35.1% of those receiving apremilast. Similarly, sPGA 0/1 was achieved by 53.6% versus 32.1%. Both efficacy endpoints, PASI 75 and sPGA 0/1, continued to improve through week 24 and remained superior with deucravacitinib [28]. Greater improvement was also observed in quality-of-life outcomes: at week 16, more patients treated with deucravacitinib achieved a DLQI score of 0/1, indicating no or minimal impact of the disease on daily life [28].

In the POETYK PSO-2 study, the comparison between deucravacitinib and apremilast showed that at week 16, PASI 75 was achieved by 53% of patients treated with deucravacitinib, compared with 39.8% of those receiving apremilast. An sPGA score of 0/1 was achieved by 49.5% of patients receiving deucravacitinib versus 33.9% of those receiving apremilast. A greater proportion of patients also achieved a DLQI score of 0/1 at weeks 16 and 24 when treated with deucravacitinib [117]. Data comparing the efficacy of deucravacitinib and apremilast in the PSO-1 and PSO-2 trials are presented in Table 7.

A pooled analysis of data from the PSO-1 and PSO-2 trials also allowed for evaluation of the safety of deucravacitinib in comparison with apremilast and placebo [107,117]. Over 52 weeks, the overall incidence of adverse events (AEs) and the exposure-adjusted incidence rate (EAIR) per 100 patient-years were similar between deucravacitinib and apremilast. A higher proportion of patients receiving apremilast, however, discontinued treatment due to adverse events. Comparative safety data are summarized in Table 8.

The most common AEs with deucravacitinib were nasopharyngitis and upper respiratory tract infections, whereas nausea, diarrhea, and headache were most frequent among patients treated with apremilast [107].

#### 5.4.2. Comparison with Biologic Therapies

Biological therapies represent a major treatment option in psoriasis, targeting key components of the immune system responsible for disease development. Their aim is to inhibit T-lymphocyte proliferation and to neutralize cytokines involved in the pathophysiology of psoriasis [109].

To compare TYK2 inhibitors such as deucravacitinib with adalimumab, a biologic agent classified as a TNF-alpha inhibitor, two long-term psoriasis treatment trials were analyzed: POETYK PSO-LTE and the REVEL extension study. Deucravacitinib is administered orally, whereas adalimumab is given subcutaneously [109].

An indication comparison was performed by matching patient characteristics from one study to the population of the other. The primary outcome was the proportion of patients achieving PASI 75 at week 112 after randomization. Secondary outcomes included PASI 75 at week 52 and PASI 90 at weeks 52 and 112 [109]. After adjusting for differences in baseline patient characteristics, the analysis showed that adults with moderate to severe psoriasis treated with deucravacitinib achieved a significantly higher PASI 75 response at week 112 compared with patients treated with adalimumab. The efficacy of adalimumab and deucravacitinib was similar up to week 52; however, deucravacitinib maintained its effectiveness more consistently over time (weeks 52–112). This may be due to the loss of efficacy of biological therapy, for example, due to the development of anti-drug antibodies [109]. At week 112, PASI 75 was achieved by 67.2% of patients treated with deucravacitinib compared with 54.0% of those receiving adalimumab. For PASI 90, the rates were 41.3% and 34%, respectively. These findings highlight the superiority of deucravacitinib at a dose of 6 mg daily compared with adalimumab at a dose of 40 mg administered subcutaneously every other week [109].

A comparison was also performed between the VOYAGE 1 trial, which evaluated adalimumab, and the POETYK PSO-1 trial, which evaluated deucravacitinib. The analysis assessed differences in the proportion of patients who achieved a score of 0/1 on the Scalp-Specific Physician’s Global Assessment (ss-PGA) scale, reflecting the condition of the scalp skin. The comparison was conducted at weeks 16, 24, and 48 of treatment. The analysis showed that a higher proportion of patients treated with deucravacitinib achieved an ss-PGA score of 0/1 compared with those treated with adalimumab. The unadjusted rate at week 48 was 73% for deucravacitinib (after correction) and 60.5% for adalimumab. A similar pattern was observed at both week 24 and week 16 of treatment. These findings demonstrate that deucravacitinib is more effective than adalimumab in the treatment of scalp psoriasis [123].

A systematic review of pharmacological treatments compared all systemic medications used for moderate to severe psoriasis, as well as those in phase 2/3 clinical development in 2022, including deucravacitinib and adalimumab. The review encompassed 140 studies with a total of 54,815 randomized participants. The results were based on a network meta-analysis. At the class level, the analysis showed that biological therapies achieved better PASI 90 outcomes compared with non-biological treatments. Infliximab demonstrated the highest efficacy (high-certainty evidence), with adalimumab ranking 11th and deucravacitinib 12th [32]. The meta-analysis indicated greater efficacy of adalimumab compared with deucravacitinib in achieving PASI 90 and PASI 75.

To compare these treatments, the Surface Under the Cumulative Ranking Curve (SUCRA), a measure indicating how well a drug ranks in terms of efficacy or safety in network meta-analyses, was applied. The SUCRA for PASI 90 was 48.7% for adalimumab and 42.5% for deucravacitinib. For PASI 75, the values were 51.3% for adalimumab and 34% for deucravacitinib, indicating that adalimumab is more effective according to this meta-analysis. However, for serious adverse events (SAEs), the SUCRA was 36.9% for adalimumab and 58.2% for deucravacitinib, indicating that deucravacitinib has a substantially better safety profile.

Moreover, the authors of the meta-analysis noted that their confidence in treatment estimates for PASI 90 was high or moderate for biological therapies but low for comparisons involving non-biological drugs, including deucravacitinib. This was due to risk of bias, imprecision, or both factors [32].

Overall, studies indicate that deucravacitinib is at least as effective as adalimumab and is associated with a more favorable safety profile.

### 5.5. Oral Administration: Advantages, Convenience, and Practical Considerations

#### 5.5.1. Patient Comfort and Preference

The selection of systemic therapy depends, among other factors, on patient preference. Injectable treatments are associated with pain, discomfort, and anxiety [17,26,124]. Some patients may be afraid to inject themselves or may need to rely on others for help, which reduces their independence. Injectable treatments also come with practical inconveniences, such as the need for refrigeration. In addition, regular ambulatory monitoring is perceived by patients as burdensome, and injection sites may lead to scarring or discoloration. All these factors contribute to the fact that some patients choose oral therapies despite the need for daily dosing [17].

Patients view oral treatment as convenient and consider it less stressful than injections. Oral therapies cause less anxiety and may interfere less with everyday life. Studies have also shown that patients currently receiving injectable therapies are more willing to start a new treatment if it is taken orally compared with patients who are already using oral medications. This demonstrates that patients tend to prefer taking a daily pill over receiving repeated injections [26].

#### 5.5.2. Impact on Adherence and Treatment Outcomes

The convenience and ease of oral administration contribute to higher treatment adherence [124]. Injection-related anxiety is also a key factor associated with poor adherence [26]. Adherence to medical recommendations is essential for therapeutic success; therefore, patient preferences should be carefully considered when planning treatment. Clinicians should take into account the treatment characteristics most relevant to the patient, their level of injection-related anxiety, convenience of use, and potential adverse effects [17,26]. An individualized treatment plan, shared decision-making, and patient education about safety and efficacy are crucial [17].

Long-term therapies, such as those used in psoriasis, are challenging for patients to maintain. In particular, after the third year of treatment, a higher rate of treatment discontinuation may be observed. It should also be noted that biologic therapies may lose their durability; by the fifth year of treatment, loss of response may reach up to 70% [24]. Therefore, strict adherence to medical recommendations and high patient compliance are key to achieving both efficacy and safety in psoriasis therapy [17].

### 5.6. Bridging the Gap: Combining High Efficacy with Oral Convenience

There are many therapeutic options for psoriasis, ranging from topical treatments for mild disease to systemic therapies for moderate to severe psoriasis. Conventional systemic therapies, apremilast, and biological agents are commonly used. However, each treatment class has its limitations. Biological therapies, although highly effective, require parenteral administration via injection and may lose efficacy over time. Therefore, there remains a need for a therapy that is effective, safe, and convenient for patients, ultimately supporting long-term adherence [24,32,66,109].

In response to these unmet needs, a new class of medications has emerged, TYK2 inhibitors such as deucravacitinib, which demonstrate high efficacy, as shown in the POETYK PSO-1, PSO-2, and long-term POETYK LTE studies [28,66,107,111,115,116,117], as well as in other trials investigating envudeucitinib [84,119] and zasocitinib [67]. The efficacy of deucravacitinib surpasses that of apremilast, the first-generation oral therapy for psoriasis [28,117], and is comparable to that of biological agents [32]; in some analyses, it may even exceed that of certain biologics, such as adalimumab [109,123].

Deucravacitinib has also been shown to be safe and well-tolerated [66,107,115,117]. Due to its convenient oral administration, unlike biological therapies requiring injection, combined with its favorable efficacy and safety profile, deucravacitinib represents an attractive therapeutic option for patients with moderate to severe psoriasis.

### 5.7. Potential Challenges and Limitations of TYK2 Inhibitors

According to the prescribing information, deucravacitinib carries a warning that its use may increase the risk of infections and should be avoided in cases of active or serious infection. Before initiating treatment, patients should be screened for tuberculosis (TB) and viral hepatitis, which is not required, for example, before starting apremilast [110,125].

Moreover, patients receiving deucravacitinib should be monitored for symptoms of tuberculosis (TB). Before initiating treatment, it is also recommended to consider administering all age-appropriate vaccinations and to avoid live vaccines during therapy [110]. Clinical data assessing a potential association between deucravacitinib exposure and the development of malignancies are limited; therefore, the risks and benefits of treatment should be carefully weighed [110,125].

The drug may cause laboratory abnormalities, such as increased creatine kinase, elevated liver enzymes, reduced GFR, and increased lipid levels [110]. Renal impairment does not require dose adjustment, but the drug should be avoided in patients with severe hepatic impairment [125]. Another potential drawback is the need for daily oral dosing, compared with biologic therapies that may allow for injections every 2–3 months [126].

There are also no comprehensive data on the use of deucravacitinib during pregnancy, nor on its presence in human milk, effects on the breastfed infant, or its impact on milk production. The benefits of breastfeeding for the infant and the clinical benefits of deucravacitinib therapy for the mother should be carefully considered [126].

Overall, oral deucravacitinib demonstrates good efficacy and safety in clinical trials. However, limited real-world experience and insufficient long-term research mean that its exact place in the psoriasis treatment landscape is still being defined.

## 6. Translational Relevance: Future Perspectives and Research Directions

Advances in stratified and personalized medicine are creating new opportunities for the precise selection of therapies in patients with psoriasis, a disease with variable course and comorbidities. Despite the availability of effective targeted treatments, some patients still exhibit a primary non-response or secondary loss of efficacy, highlighting the need for a better understanding of disease mechanisms and individual factors that influence treatment outcomes.

The introduction of selective TYK2 inhibitors, such as deucravacitinib, represents an important step toward more precise and safer treatment, as well as the goal of achieving durable remission. However, fully realizing the potential of this new class of drugs in routine clinical practice requires addressing the remaining evidence gaps [127,128,129].

### 6.1. Current Evidence Limitations and the Need for Real-World Data

Despite promising results from clinical trials and the rapid development of the TYK2 inhibitor class, precisely positioning deucravacitinib within therapeutic algorithms remains a challenge.

Solid comparative data are currently available for apremilast; however, a key limitation is the lack of head-to-head studies with newer biologics, such as IL-17 and IL-23 inhibitors.

Such studies are essential to clearly determine which patients may benefit from oral therapy as an equivalent alternative to injectable treatment, and in which cases initiating biological therapy should remain the priority.

Real-world evidence (RWE) should also serve as an essential complement to clinical trials. Extensive retrospective analyses are needed to confirm the efficacy and safety of the drug in populations often excluded from standard trials.

Future analyses should additionally focus on evaluating the durability of response after discontinuation (time to relapse) and drug survival, which will enable further optimization of therapeutic strategies [130,131,132,133].

### 6.2. Next-Generation TYK2 Inhibitors

The success of deucravacitinib has confirmed that kinase regulatory domains can serve not only as effective but also as exceptionally safe therapeutic targets. This discovery has paved the way for future treatment strategies focused on the precise modulation of inflammatory pathways, which are central to the pathogenesis of psoriasis and other immune-mediated diseases [75,107,134].

Among TYK2 inhibitors in advanced clinical development, zasocitinib (TAK-279) and envudeucitinib (ESK-001) are particularly notable, having demonstrated promising potential in achieving high clinical response rates in phase 2/3 trials for the treatment of moderate to severe plaque psoriasis. Nevertheless, their efficacy and safety require further confirmation in larger, subsequent studies [67,84,120].

The group of investigated compounds is further complemented by D-2570, a selective TYK2 inhibitor, which demonstrated high clinical efficacy and a favorable safety profile in a phase 2 study involving patients with moderate to severe plaque psoriasis. Additional research is needed to confirm these findings [135].

QL-1200186, a novel allosteric TYK2 kinase inhibitor, is currently in early development. Preclinical data suggest that this molecule effectively inhibits cytokine-dependent inflammatory responses and may provide even greater selectivity than deucravacitinib. Despite promising results in preclinical models, its safety and efficacy need to be confirmed in clinical trials to establish its potential for treating psoriasis and other immune-mediated diseases [136].

### 6.3. Unlocking Treatment Opportunities for Diverse Patient Populations

The favorable safety profile and specific mechanism of action of selective TYK2 inhibitors expand personalized treatment options in psoriasis. This is particularly relevant for older adults, pediatric patients, and those with comorbidities such as diabetes, obesity, or hypertension, as well as patients with intolerance to previous treatments.

Furthermore, the potential of this class of drugs is currently being evaluated in studies involving adolescents (12–18 years), which may enable the future introduction of modern oral therapies for the pediatric population.

Dedicated clinical trials are necessary to fully assess the long-term benefit–risk profile in these specific patient populations [66,131].

### 6.4. Exploring New Therapeutic Indications

Expanding the patient population also opens the prospect of investigating new therapeutic indications for selective TYK2 inhibitors in immune-mediated inflammatory diseases (IMIDs). The role of TYK2 kinase in signaling proinflammatory cytokines, which are central to chronic inflammation, supports investigating this therapeutic pathway in additional indications.

Preliminary data indicate that deucravacitinib has a favorable efficacy and safety profile in patients with psoriatic arthritis (PsA). Further phase III studies, including head-to-head comparisons, are needed to confirm its safety and clinical benefits and to clearly define the drug’s place within treatment algorithms for this condition.

In addition to psoriasis and psoriatic arthritis, the efficacy of deucravacitinib and other TYK2 inhibitors is being evaluated for the treatment of several immune-mediated conditions, including cutaneous lupus erythematosus (CLE), systemic lupus erythematosus (SLE), Sjögren’s syndrome, dermatomyositis, inflammatory bowel disease, uveitis, and hidradenitis suppurativa [137,138,139,140,141,142,143,144,145].

### 6.5. Towards Precision Medicine: Biomarkers for Predicting Treatment Response

The ability to predict treatment response remains a key unmet need in the management of psoriasis. Identifying reliable biomarkers (genetic, molecular, serological) is key to personalized medicine, allowing for patient stratification and the selection of therapies suited to individual needs.

Particularly valuable would be the preventive identification of individuals who are more likely to achieve a durable and safe response to a given treatment, thereby avoiding ineffective therapies and optimizing clinical outcomes.

Previous studies have primarily focused on biological treatments, with the HLA-C*06:02 allele being the most frequently analyzed genetic biomarker, correlating both with susceptibility to psoriasis and with differential responses to selected biologic therapies [128,146,147,148].

For TYK2 inhibitors, preliminary data exist on molecular and serological markers, including IL-23/Th17 pathway cytokine levels, which correlate with disease activity. Effective treatment with deucravacitinib has been associated with reductions in these mediators, although these findings require confirmation in larger studies.

Up to now, no reliable predictive biomarkers with proven clinical utility have been identified for TYK2 inhibitors, underscoring the need for further research in this area [79,80,128].

## 7. Conclusions

The introduction of selective, allosteric TYK2 kinase inhibitors marks a new chapter in the treatment of plaque psoriasis. These drugs effectively bridge the therapeutic gap between conventional oral medications, which often fail to provide adequate disease control and carry a risk of toxicity, and highly effective biologic therapies, which also have limitations, including their injectable route of administration, perceived as invasive, as an often painful treatment option.

Deucravacitinib’s unique mechanism of action, which stabilizes the pseudokinase domain, effectively locks the enzyme in its inactive state. This mechanism avoids the systemic toxicity seen with non-selective JAK inhibitors. It offers an optimal balance of clinical efficacy, oral convenience, and a favorable safety profile.

These properties allow for a more personalized approach to treatment and enhance patients’ quality of life. This is particularly important given the chronic and long-term nature of the disease, which requires carefully tailored therapy with minimal toxicity. Combined with the convenient oral dosage-form therapy, this promotes better treatment adherence.

These features underscore the increasing importance of TYK2 inhibitors in clinical practice and their potential to become the preferred first-line oral systemic therapy, effectively supplanting older conventional medications in the treatment of moderate to severe psoriasis. They may also offer an attractive option for patients who did not respond to prior biologic therapies.

**Figure 4:**
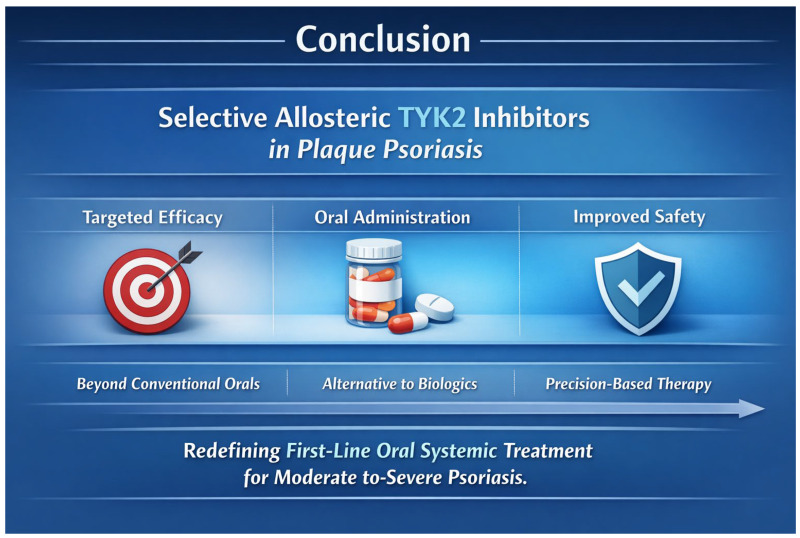
Activation of Janus Kinase and comparison of non-selective orthosteric JAK inhibition versus selective allosteric TYK2 inhibition.

In the era of evidence-based medicine, this class represents a scientifically grounded option for psoriasis management, with the potential to shape future therapeutic strategies, optimize patient care, and expand the landscape of precision systemic therapies. Figure 4 provides a summary of the key clinical advantages and emerging therapeutic role of selective allosteric TYK2 inhibitors.

## Figures and Tables

**Table 1:** Exposure-Adjusted Incidence Rates (EAIRs) of adverse events for deucravacitinib: year 1 vs. 3-year cumulative.

Adverse Event (AE)/Category	Through Year 1 (EAIR per 100 Person-Years)	3-Year Cumulative (EAIR per 100 Person-Years)	Clinical Interpretation
General Tolerability			
AEs total	229.2	144.8	↓ Decreased
Discontinuation due to AEs	4.4	2.4	↓ Decreased
Most Common Adverse Events (EAIR per 100 person-years ≥ 5)			
Nasopharyngitis	26.1	11.4	↓ Decreased
Upper respiratory tract infection	13.4	6.2	↓ Decreased
COVID-19	0.5	8.0	↑ Increased (Increase driven by the pandemic peak during LTE trial.)
Headache	8.5	3.4	↓ Decreased
Arthralgia	5.7	3.3	↓ Decreased
Diarrhea	7.3	3.0	↓ Decreased
Adverse Events of Interest (AEIs)			
Serious Infections (excl. COVID-19)	1.7	0.9	=Stable
Herpes zoster	0.8	0.6	=Stable
MACE (major adverse cardiovascular events)	0.3	0.3	=Stable
VTE (venous thromboembolism	0.2	0.1	=Stable
Malignant diseases	1.0	0.9	=Stable
Skin events of interest			
Acne	2.9	1.3	↓ Decreased
Folliculitis	2.8	1.1	↓ Decreased

**Table 2:** Clinical efficacy of deucravacitinib at week 12 by orally administered dose.

Treatment Group/Dose	Dosing Schedule	PASI 75 at Week 12
Placebo	-	7%
3 mg	Every other day	9%
3 mg	Once daily	39%
3 mg	Twice daily	69%
6 mg	Twice daily	67%
12 mg	Once daily	75%

**Table 3:** PASI 75 and sPGA 0/1 responses at week 16 in the POETYK PSO-1 and PSO-2 trials.

Outcome (Week 16)	Deucravacitinib	Apremilast	Placebo
POETYK PSO-1			
PASI 75	58.4%	35.1%	12.7%
sPGA 0/1	53.6%	32.1%	7.2%
POETYK PSO-1			
PASI 75	53.0%	39.8%	9.4%
sPGA 0/1	49.5%	33.9%	8.6%

**Table 4:** Clinical efficacy of envudeucitinib at week 12 by orally administered dose.

Treatment Group/Dose	Dosing Schedule	PASI 75	sPGA 0/1
Placebo	-	0%	8%
10 mg	Once daily	19%	17%
20 mg	Once daily	33%	39%
40 mg	Once daily	56%	49%
20 mg	Twice daily	56%	54%
40 mg	Twice daily	64%	59%

**Table 5:** Clinical efficacy of envudeucitinib at weeks 28 and 52 by orally administered dose.

Treatment Group/Dose	Dosing Schedule	PASI 75	sPGA 0/1
Placebo	-	-	-
40 mg	Once daily (week 28)	67.5%	51.3%
40 mg	Twice daily (week 52)	77.5%	61.3%
Overall, 40 mg	Twice daily (week 52)	77.8%	59.7%

**Table 6:** Clinical efficacy of zasocitinib at week 12 by orally administered dose.

Treatment Group/Dose (Once Daily)	PASI 75 at Week 12	sPGA 0/1 at Week 12
Placebo	6%	4%
2 mg	18%	10%
5 mg	44%	27%
15 mg	68%	49%
30 mg	67%	52%

**Table 7:** Comparison of the efficacy of deucravacitinib and apremilast at weeks 16 and 24 in the PSO-1 and PSO-2 trials.

POETYK PSO-1		
	Deucravacitinib	Apremilast
PASI 75		
Week 16	58.4%	35.1%
Week 24	69.3%	38.1%
PASI 90		
Week 16	35.5%	19.6%
Week 24	42.2%	22.0%
PASI 100		
Week 16	14.2%	3.0%
Week 24	17.5%	6.5%
sPGA 0/1		
Week 16	53.6%	32.1%
Week 24	58.7%	31.0%
	sPGA 0	
Week 16	17.5%	4.8%
Week 24	18.1%	6.5%
DLQI 0/1		
Week 16	41.0%	28.6%
Week 24	48.1%	24.2%
**POETYK PSO-2**		
	**Deucravacitinib**	**Apremilast**
	PASI 75	
Week 16	53.0%	39.8%
Week 24	58.7%	37.8%
PASI 90		
Week 16	27.0%	18.1%
Week 24	32.5%	19.7%
PASI 100		
Week 16	10.2%	4.3%
Week 24	13.1%	6.7%
sPGA 0/1		
Week 16	49.5%	33.9%
Week 24	49.8%	29.5%
sPGA 0		
Week 16	15.7%	6.3%
Week 24	17.1%	7.9%
DLQI 0/1		
Week 16	37.6%	23.1%
Week 24	41.4%	21.5%

**Table 8:** Comparison of adverse events between deucravacitinib and apremilast.

Type of Adverse Event	Deucravacitinib		Apremilast	
	% of Patients	EAIR/100 PY	% of Patients	EAIR/100 PY
Any AE	72.9	217.4	70.9	281.1
SAE	4.0	5.7	2.1	4.0
AE leading to discontinuation	3.2	9.3	6.2	11.6

## Data Availability

Not applicable.
